# Qualitative evaluation of the Rehabilitation Exercise and psycholoGical support After COVID-19 InfectioN (REGAIN) randomised controlled trial (RCT): ‘you are not alone’

**DOI:** 10.1136/bmjopen-2024-085950

**Published:** 2025-01-29

**Authors:** Kate Seers, Vivien P Nichols, Julie Bruce, Stuart Ennis, Peter Heine, Shilpa Patel, Harbinder Kaur Sandhu, Martin Underwood, Gordon McGregor, on behalf of our REGAIN collaborators., Bartholomew Sheehan

**Affiliations:** 1 Warwick Medical School, University of Warwick Medical School, Coventry, UK; 2 Warwick Clinical Trials Unit, University of Warwick, Coventry, UK; 3 University Hospitals Coventry and Warwickshire NHS Trust, Coventry, UK; 4 Warwick Medical School, University of Warwick, Coventry, UK; 5 Cardiac rehabilitation, University Hospital Coventry, Coventry, UK; 6 CTU, Warwick Medical School, Coventry, UK; 7 Cardiopulmonary, University Hospitals Coventry and Warwickshire NHS Trust, Coventry, UK; 8 WMS, Clinical Trials Unit, University of Warwick, Coventry, UK; 9 Clinical Trials Unit, University of Warwick Warwick Medical School, Coventry, UK; 10 Cardiac Rehabilitation, University Hospitals Coventry and Warwickshire NHS Trust, Coventry, UK

**Keywords:** Qualitative Research, COVID-19, Post-Acute COVID-19 Syndrome, Rehabilitation Medicine

## Abstract

**Abstract:**

**Background:**

This qualitative evaluation was embedded in the Rehabilitation Exercise and psycholoGical support After COVID-19 InfectioN (REGAIN) study, a randomised controlled trial (RCT) for those with post-COVID-19 condition (‘long COVID’) after hospital admission for COVID-19, comparing weekly home-based, live online supervised group exercise and psychological support sessions with ‘best practice usual care’ (a single session of advice).

**Objective:**

To increase our understanding of how and why the REGAIN programme might have worked and what helped or hindered this intervention.

**Design:**

A qualitative evaluation which utilised interviews with participants and practitioners delivering the intervention. Framework and thematic analysis were used to analyse the findings.

**Setting:**

England and Wales, UK.

**Participants:**

Adults discharged from National Health Service (NHS) hospitals at least 3 months previously after COVID-19, with ongoing physical and/or mental health sequelae.

**Results:**

Twenty intervention participants, 20 control participants and five practitioners were interviewed.

The themes from the group support sessions were: (1) you are not alone; (2) sharing experiences and addressing worries; (3) gaining new perspectives; (4) hope for progression; (5) peer support and bonding; (6) integration of facilitation skills; (7) modified activity pacing and goal setting, and (8) giving participants structure. The themes from group exercise were: (1) monitoring and modification of the online exercise; (2) catering for differing abilities; (3) feeling safe and confident to exercise; (4) progression of fitness; (5) optimal timing in the recovery trajectory; (6) group effect; (7) initial apprehension about exercise group; (8) gauging exercise capabilities; (9) translating exercises into life; and (10) on-demand supplementary videos. The 1:1 consultation sessions revealed patients needed to tell their stories.

**Conclusion:**

Being listened to and being understood by someone ‘who got it’ was very important to people with post-COVID-19 condition. The group sessions of both exercise and psychological support were valued by participants, working together, and learning from each other in the face of a new disease within a global pandemic.

**STRENGTHS AND LIMITATIONS OF THE STUDY**

- This qualitative evaluation explored feasibility and acceptability, and the sample was purposively determined, ensuring a diverse range of views and experiences would be documented.
- Interviewing participants in both arms of the study as well as the practitioners delivering the intervention allowed for a more detailed understanding of this study and what it was like to take part.
- The design of this qualitative evaluation did not allow for detailed collection of data (eg, interviews) from non-attendees or those who dropped out.
- Participants had been hospitalised with COVID-19 which may limit the generalisability of findings to the whole post-COVID-19 condition population.
- Although a range of interviewee characteristics was aimed for, 75% of participants in this qualitative evaluation were white; thus, the reach of the study was potentially limited.

## Background

 More than 17 million people may have experienced COVID-19 symptoms lasting more than 4 weeks,^1 2^ with 1.3 million in the UK having symptoms beyond 1 year.^3^ The term ‘long COVID’, now commonly used as an alternative to post-COVID-19-condition, was first used by patients in online chat groups who were linking up as survivors of this virus experiencing debilitating symptoms.^4^

The Rehabilitation Exercise and psycholoGical support After COVID-19 InfectioN (REGAIN) study was a multicentre randomised controlled trial (RCT) involving adults discharged from hospital more than 3 months previously after an admission for COVID-19 (n=585) who were still experiencing ongoing physical or mental health sequalae.^5 6^ The study tested home-based, live supervised group exercise (eight sessions) and psychological support (six sessions), delivered online over 8 weeks by clinical exercise physiologists or physiotherapists trained in the REGAIN intervention^7^ compared with ‘best practice usual care’ which consisted of a 30-min, online 1:1 consultation with a REGAIN practitioner and a booklet with components of the National Health Service (NHS) England ‘Your COVID recovery’ resource.^8^ The intervention group also had a 1 hour 1:1 online session, including familiarisation with the online environment. Overall, the REGAIN intervention was found to generate a modest but clinically meaningful improvement in quality of life as measured by the Patient-Reported Outcomes Measurement Information System (PROMIS) Preference (PROPr) score (0.03 (95% CI 0.01 to 0.05), scale range −0.22 to 1.0) at 3 months, the primary outcome, when compared with best practice usual care.^6^

To contextualise the clinical findings of the REGAIN study, we conducted an embedded qualitative evaluation to investigate feasibility and acceptability. The aim was to better understand participant and practitioner experience and any barriers or enablers to taking part to inform any implementation of the intervention in clinical practice.

## Methods

This qualitative evaluation involved interviews with 20 intervention and 20 control participants and five practitioners who delivered the intervention from a single, central trial delivery hub (Atrium Health, a not-for-profit social enterprise delivering rehabilitation services, under sub-contract with University Hospitals of Coventry and Warwickshire NHS Trust).

This number of interviews was selected to ensure we went beyond code saturation and reached meaning saturation.^9^ We identified a purposive sample of participants to ensure a range of experiences based on: trial allocation, sex, ethnicity, age, level of hospital care (Intensive Care Unit/High Dependency Unit or ward), severity of post-traumatic stress disorder (PTSD) symptoms (Impact of Event Scale-6 (IES-6))^10^ and severity of mood symptoms on the Hospital Anxiety and Depression Scale (HADS),^11^ all collected as part of the main RCT.

Participants were contacted if they had agreed to be approached for an interview on their main trial consent form. We sent an email or text (depending on the stated preference for contact) with a link to the interview participant information sheet; a following link directed participants to an online informed consent form. On completion, the qualitative researcher received an email confirmation of consent and telephoned the participant to arrange an interview. All interviews were carried out remotely using MS Teams or by telephone if preferred, by a very experienced female physiotherapist research fellow with an MSc by Research (VN). No other people who were non-participants were involved in the interview. We recorded and checked consent separately and then recorded the interview. Interviews were transcribed by a university-approved transcription service. All data were held in a digitally secure environment with limited access. Transcriptions were checked for accuracy by VN, and NVivo software was used to organise and explore the data.

Interview guides were developed by the research team (KS and VN) to include questions about experiences of being in the study. These were reviewed by the ethics committee as part of the ethical approval process and were pilot tested with eight participants, with no major changes being required (see online supplemental appendix 1). No repeat interviews were carried out. Brief field notes were kept, written up as soon as possible after the interview. Transcripts were not sent to participants for comment. A relationship was established with participants during the consent procedure. There was no relationship before the study started. The participants were aware that VN was part of the study team. She had previous experience in undertaking qualitative evaluations.

### Analysis

We used framework analysis to analyse the interviews.^12^ This enabled us to understand what had happened during the study and if and how participants had used the interventions. This involved data familiarisation (listening to recordings, reading transcripts and reading field notes). We did not use a coding tree because we used the structure from the interview schedule, which is presented in the findings. Using framework analysis meant we used the interview schedule to guide the structure for the analysis, but we were also vigilant for any newly emerging codes not previously included in the framework. Further thematic analysis^13^ was used to explore participant experiences of their post-COVID-19 condition. The thematic analysis involved reading through the transcripts and identifying patterns in the meaning across the data set. The thematic analysis was undertaken by VN and KS who met regularly to code, review and discuss emerging themes. Emerging themes were not discussed with participants. We found data saturation was reached by 20 interviews, where we had gone beyond code saturation (heard it all) and had reached meaning saturation (understood it).^9^

### Patient and public involvement

Our patient partner working group drove the concept for the overall study and grant funding application early in the COVID-19 pandemic. Patient partners were involved as co-applicants in the grant funding application. On receipt of the award, our patient and stakeholder working group were integral to the rapid design, co-creation and pilot testing of the REGAIN intervention and trial processes.^7^ Subsequently, patient partners participated as members of the trial management group and trial steering committee. Thus, there was patient and public involvement when interpreting the findings.

## Results

The REGAIN RCT recruited from January 2021 to July 2022. Qualitative evaluation data collection took place for participants from April 2021 to November 2022 and from November 2022 to December 2022 for practitioner interviews.

### Participant characteristics

We used purposive sampling to ensure a diverse range of participants, which was appropriate given the ‘newness’ of post-COVID-19 condition; the demographic affected was diverse, and symptoms were many and varied. We interviewed 40 participants, 20 control and 20 intervention. Participants were aged 32–80 years, mean 51 years (table 1).

**Table 1:** Participant characteristics

Characteristics	Participants (n=40)
Sex	
Male	20
Female	20
Ethnicity	
White	30
Ethnic minority background	9
Prefer not to say	1
Age	
<65 years	28
≥ 65 years	12
Hospital care setting	
Ward	20
Intensive Care/High Dependency Units	20
IES-6 scores*	
<11	15
≥11	25
Hospital anxiety and depression scores*	
Anxiety <11	27
Anxiety ≥11	13
Depression <11	29
Depression ≥11	11

* Baseline scores before randomisation. Impact of Event Scale-6 (IES-6) (Impact of Event) score equal to or more than 11, and Hhospital Aanxiety and Ddepression scores equal or more than 11 were threshold for possible case level mental health disorder.

The interviews lasted between 19 min and 1 hour and 14 min, median 38.5 min. In addition to the 20 intervention and 20 control participants, 18 people who said in the main RCT that they were happy to be approached about an interview decided not to be interviewed. Fifteen of these 18 people were in the control group.

### Practitioners

Eight practitioners delivered the intervention. The lead intervention designer/practitioner was not interviewed, but the other seven were invited for interview after all the interventions were completed. Two were unable to be interviewed due to work constraints, having moved on from their REGAIN role. Interviewees were given numerical identifiers for anonymity. Practitioner interviewees were clinical exercise physiologists or clinical exercise specialists with between 4 years to over 10 years of experience in that role.

### Findings

Identifiers following quotations are *Int* for patient participants and *P* for practitioners.

A table listing all the themes and whether they were from participant or practitioner interviews is presented in online supplemental table S1.

### Regain intervention group

The relationship between the participants and practitioners appeared to be one of mutual respect, working together and learning from each other in the face of a new disease within a pandemic.

*…Effectively they come for the group support and they stay for the exercise not the other way around*. P2

*…And I think if somebody said to me if you would say to me, ‘…we haven’t got the resources to do the exercise and the support. What would you rather do? I would go for the support every time*. Int13

Given that the support sessions were seen as important, these themes are presented first.

### Support session themes

1. You are not alone.
2. Sharing experiences and addressing worries.
3. Gaining new perspectives.
4. Hope for progression
5. Peer support and bonding.

These five themes came from both patient and practitioner interviews. In addition, the following three themes emerged only from the practitioner interviews:

1. Integration of facilitation skills.
2. Modified activity pacing and goal setting.
3. Giving participants structure.

These will now be presented in more detail with supporting quotations.

#### You are not alone

People recognised the positive effect of getting together, although virtually, which made people feel that they were not alone in what they were experiencing. They often did not know anyone else with post-COVID-19 condition.

…*Talking to others with similar experiences helps, and you’re thinking, ‘You’re not alone with these, sort of, thoughts of hospital treatments and what it was like, and…the fatigue and dealing with it, so those were probably the two key things for me at the session*. Int9

*I enjoyed it because I was able to see what other people were going through and not feel like I was the only one going through it, you know, and then how others were dealing with it*. Int20

#### Sharing experiences and addressing worries

Sharing experiences was valued by most and practitioners also felt this was a key component of REGAIN. Elements of validation and normality could be found through conversations which gave an opportunity to air fears and worries in a supportive environment.

…*Everyone helped to motivate everybody else…And people were really open about the things that they had struggled with*… Int29

…*Actually for me, honestly, the best, best thing was talking to other people and learning about their experiences and actually knowing how similar actually our experiences were*… Int17

#### Gaining new perspectives

Practitioners suggested strategies they might want to explore during the support sessions, and some participants spoke of looking at things in a different way or deciding to make positive changes for themselves as a result of their discussions.

*I think there was some that were more challenging than others and more emotional, I think,‘cause one of the things we talked about was unhelpful thoughts… All we’re saying is, ‘Here are some coping strategies that might help,’ and that’s the keywords, they might help. No one’s saying these will sort it*…P3

*So I feel much more confident in myself and my self-esteem has got, has risen. Because I think the support sessions made me realise, when you are talking to other people, is that actually we have gone through so much and why we shouldn’t be proud of whatever condition we are in now, you know*. Int36

#### Hope for progression

Some participants spoke about improvements they could make to help their recovery, and practitioners could often see these progressions.

…*Goal setting was so good for people because they could actually see their improvement then. And I know that there was quite a few actually achieved their goal, achieved their initial Goals as they were going through the programme*. P5

….*Having that support in those groups and seeing how other people cope with things and have progressed was really helpful ‘cause you’re just like ‘Well if they can do it, why can’t I?* Int36

#### Peer support and bonding

Group sharing and bonding were in evidence in all the groups, where people often compared themselves to others and they enjoyed discussions with people ‘in the same boat’. Participants sometimes kept in contact with other group members after the intervention had finished.

T*he main thing that I’ve noticed that people really benefit from being able to share their experiences…it’s actually the peer support that they probably found most useful. But that was then the springboard for people to want to improve their fitness*. P2

*We’re getting different ideas off each other in the groups. It enabled them to obviously progress, they got different ideas…they had that bank of support off people within the group*. P5

*Actually, we have actually swapped emails and we’ve had a Zoom between us which is really nice…like anything, anybody who’s been through a particular trauma….nobody really knows what you’ve been through and only the people who’ve been through it know what you’ve been through*…Int13

These next three themes came from practitioners only.

#### Integration of facilitation kills

Practitioners spoke about specific facilitation skills they had been taught and used such as giving participants time to respond with short spaces of silence. Practitioners felt they were learning all the time and were happy to let the group run with guidance and open questions based around the video clips and guidance sheets.

*I felt like a lot of them are like, ‘Well, you’ve not had long COVID so you don’t get it’…The examples, the video and these sorts of things and reflecting back onto them like you guys like we're learning from you, this is like more of a like what we're learning from you and you guys can learn from each other. I'm just here to help you, you discuss it through. So I think it got better with that*. P1

#### Modified activity pacing and goal setting

Pacing was needed and used by almost everyone in response to changing energy levels. Goal setting was used effectively by some, but there was a need to stipulate that these weren’t necessarily activity based.

*And so that that traditional goal setting model didn't really fit that…I found myself having to be a bit more nuanced about that as I’ve got through the groups that I, I have to say to people that we can't always set kind of physical goals because we don’t, we don’t, you know, these are not all within the locus of our control…the goals had to become less, yeah, less physical I think…it was important to have some, but not all.* P2

#### Giving participants structure

*And some people, it gave people structure as well because the people who were quite ill and were, they couldn’t get out much, they knew they’d got this group to look forward to… it’s good to sort of discuss and open up about these things and a lot of people that maybe don’t have anyone to talk to, there’s a lot of very isolated people on here that haven’t actually really spoken to anyone since the pandemic, haven’t really left the house. So in terms of getting them to open up and talk. In their own homes where they [are] comfortable, I think probably it was quite beneficial for them*. P1

The themes from the intervention exercise groups are presented next.

There were six themes across the participant and practitioner responses about the exercise groups.

1. Monitoring and modification of online exercise.
2. Catering for differing abilities.
3. Feeling safe and confident to exercise.
4. Progression of fitness.
5. Optimal timing in the recovery trajectory.
6. Group effect.

These are then followed by four themes coming only from participants.

1. Initial apprehension about exercise.
2. Gauging exercise capabilities.
3. Translating exercise into life
4. On-demand supplementary video.

These are now presented in more detail with supporting quotations.

#### Monitoring and modification of online exercise

Participants and practitioners felt online exercise classes were very different from face to face but worked better than initially expected. Practitioners had to use more ‘checking in’ with the participants using visual and speech clues to assess how the participants were managing which was noted and valued by the participants. Practitioners would also ask for frequent feedback from each participant to gain information on how to pitch the exercises at the correct level for each. The second practitioner (present in each exercise session) could also gauge this from a safety perspective. These aspects both reassured and made the participants feel less fearful.

*I think I was surprised at the level of interaction, considering it’s all, you know, electronic, and no face to face. I felt I’d had a good connection with everybody. I felt the exercises, the timer was clear on the wall, you could see how long you had to carry on for. All the exercises were demonstrated and re-demonstrated in a safe way and if you were not bending the bits that you should bend or, you’d be told….but in a positive way, positive reinforcement. So no, I think it was…I think I learnt a lot from it*. Int10

…*Throughout we would be using a talk test, so we’d be constantly asking them for, you know, ‘How are you feeling, how’s things?’ you know, ‘What you been up to this week?’* P3

…*I think it made me feel a bit more positive about wanting to go back to exercise and, like, as [name] said, you know, ‘You’re not the only one that’s going to be breathless, and if you do get breathless just step down a notch or sit it out…’ But, yeah, it, yeah, it stopped me being so afraid, really.* Int16

#### Catering for differing abilities

Participants quickly realised that everyone in the group had different physical abilities and often compared others to themselves. All the practitioners spoke of the participants as presenting in a way which was like nothing they had seen before with regard to: multimorbidity, complex symptoms including fatigue, differing abilities and ages.

*They were really good, really helpful because there was, like, different, I think [name] did two or three variants of, ‘If you want to make it easy, sit down and do it. If you want to make it a little bit harder on yourself we’ll do it like this.’ So every week there was, I knew I was using the harder one so that’s how I knew I was progressing ‘cause I could do the harder version every week. So I just think the way that they had got it set up for all different capabilities was really good*. Int16

#### Feeling safe and confident to exercise

Feeling safe to exercise overcame fear avoidance and fostered confidence to try.

*We felt cared for. I didn’t feel like we were asked to do anything that our instructor didn’t think we were capable of all the way through, which, again, I felt quite safe*. Int10

*I think the biggest impact for me was overcoming my fear of exercise in a way that was gentle and taking into consideration my physical well-being or lack of well-being. So, yes, that was the biggest help…I could see that I can manage some exercise, and I could see that it actually helps me feel more energetic*. Int37

#### Progression of fitness

Some participants felt they had progressed in differing ways.

*I think we started the gentle exercises and then we upped the pace, so probably, you know, just take that message, you start small and gradually build up. Don’t push yourself or else you’ll set yourself (up)to fail, I think. So, yes, just gentle*. Int18

*And that’s why with the group sessions every week even I thought, ‘Oh, you struggled with that the last time and I’m not as bad this time.’ So, yeah, it put my mind at ease and it made me realise, you know, don’t be scared of getting breathless ‘cause each time you do do something it is going to get better. It changed my mind-set and made me get up and do things*… Int16

#### Optimal timing in the recovery trajectory

Practitioners felt that some participants came to the tudy at the optimal time for them. Others were considered to be past the point of it being relevant or useful for them as they had recovered sufficiently to be able to return to most of their normal activities

*But REGAIN just carried on what I wanted to do, so it was good. But the thing is I had come to a point where I needed something so REGAIN was helpful.…Yes. If I was in it before I would not have done it because I could not even walk…And being able to have someone that you can reach out to is, I think it is essential at this stage, I think*. Int20

#### Group effect

Exercising in a group brought some camaraderie, support and motivation.

*On my group,there was a disabled lady and she used to do all the exercises in a chair, and there was no, nobody felt uncomfortable, nobody felt anybody, made anybody else feel uncomfortable. It was a really good group actually…and it would have been nice to have stayed in touch with them*. Int36

…*I liked the input from, and seeing the reactions from everyone else on the course as well, because it added a little bit of an extra encouragement to quite a few of us to just do a little bit more when we were seeing that other people were struggling and/or to give them a little bit more encouragement as well and motivate them to do a little bit more. And that was in the first couple of weeks and then that, that sort of filtered through to everybody, pushing everybody else. Even the ones that were struggling were saying, you know, ‘Look, just try one more’, or, ‘Go on, you can do it’, that kind of encouragement*. Int39

The final four exercise themes (themes 7 to 10) that were specific to participants and not reported by practitioners.

#### Initial apprehension about exercise

Patients reported initial apprehension and fear of exercise.

*I was quite nervous actually. I remember just logging on and, I mean, ‘cause I had already had the one-to-one with [name]…it was a familiar face. And then I remember thinking, “Can all these other people see me exercising?” and like, you know, ‘Oh, I don’t know if I’m going to enjoy this.’ And I remember I did struggle with the first one but I felt better for doing it. So, but, yeah, I was, I was quite nervous when I did the first one but then, like, as the weeks go on you just, we all got to know each other and it was just really good fun*. Int16

*I remember the first one being absolutely terrified, because I thought, ‘I’m going to run out of breath within two minutes and going to not be able to do it at all and it’s going…and I’m going to feel really ill after it.’ And but once we got started and it was very gentle and it was nothing too much, I thought, ‘Yeah, I can do this, this is okay’.* Int13

#### Gauging exercise capabilities

Participants spoke about using the exercise sessions to gauge their boundaries and physical abilities.

*I want to see if the exercise helps me, because I don’t know what exercises to do. This is all about improving you know*. Int17

*I’ve noticed like I could do things like, say I did half an hour in the garden…and I seem to be, with the exercises where it’s made me a bit more flexible it seems to be alright if I’m out there and I, it doesn’t take me so long to recover after doing something*… Int28

*My muscles generally felt more toned, because of the exercise. And I felt much more confident and encouraged that things will be getting better for me, because I could see that I can manage some exercise, and I could see that it actually helps me feel more energetic*. Int37

#### Translating exercise into life

Participants gave examples of how exercise sessions had been translated into their everyday life.

*And they’ve also given me the confidence to…So I’ve joined, or we joined, me and my partner, because I was a little bit anxious about going on my own, aqua spin classes…And I wouldn’t have had the confidence to do that if I hadn’t have been on the REGAIN programme…* Int36

*So, by the end of the classes, I realised I was actually getting up off the sofa and just carrying on and not stopping and catching my breath. So, I thought actually no they’re working. So, it gave me confidence that I could do more and push myself a little bit more…I’ve started going to the gym and it’s given me the confidence to do that. So, I’ve carried on, you know, trying to improve my fitness*. Int40

#### On-demand supplementary video

On-demand sessions did not seem to be a regular feature for interviewees. Although some did access some of the sessions, there did not seem to be anyone who regularly attended sessions over and above their weekly group session. The original REGAIN design was for participants to exercise on their own in between sessions.

### Findings from the online 1:1 sessions with a practitioner

Two main findings were evident from the 1:1 online sessions: (1) the 1:1 session with a practitioner revealed there were traumatic stories needing to be told for both control and intervention participants and (2) the importance of support for the practitioner.

#### Traumatic stories needing to be told

Although the practitioners felt prepared to deliver the intervention, nothing could have prepared them for potential traumatic/horrific accounts which some of the participants shared with them. These initial 1:1 sessions were often lengthy and could be distressing, but all practitioners felt that it was important to listen and ‘bear witness’ to these and not curtail them. Some practitioners felt it was a humbling experience and that sometimes this was the only opportunity people had had to divulge their whole story. These stories helped the practitioner to tailor the physical activity guidance as per protocol.

…*Most of it was the fact that they felt that they were listened to, but also that they were finally talking to somebody who got it, you know, as they said, because a lot of their close relations and employers just didn’t and even though they might have wanted to*. P2

…*And some of the stuff he told me he hadn’t told his family…I was bowled over by that, actually that he hadn’t shared*… P3

#### Practitioner support

Practitioners felt supported throughout by their colleagues and the wider team. They often needed a debrief after a challenging session and used the health psychologists to ask for advice when necessary. Regular catchup meetings were said to be valuable, and a camaraderie was fostered by the team all working from one central venue and working with different practitioners. Newer practitioners felt they were prepared well in their training and the support systems in place and continuity from the initial 1:1, and taking the group sessions was appreciated.

*And I felt quite emotional at the end of it and, you know, that was one of the nice things from the support side of things that the team had said,‘Just look, if ever you feel like that just give us a call and we can have chat. We can have a bit of a debrief if that’s what you need*’. P3

*It was quite draining at times. If you were doing quite a few 1:1’s kinda back-to-back, sometimes yeah. Sometimes it was quite draining. And that’s why I think it was, it was useful that the people, the people involved with the trial had all got quite a bit of experience as well about whether it’s life experience or dealing in these situations as well*. P5

### Participant perspective specific to the control group

Four themes emerged that were specific to the control group: (1) trial processes and preference of allocation; (2) experiences; (3) did REGAIN make a difference and (4) your COVID recovery resource.

1. *Trial processes and preference of allocation*: participants generally had a good idea about randomisation, and the questionnaires were seen as straightforward. Most had no preference to which group they were allocated, although five would have preferred the group intervention.
2. *Experiences*: all comments about the REGAIN practitioners were positive, that they were informative and friendly and listed to the participant. They were reassured they were on the right track, the need to go slowly with their activities, but to increase a bit if they were able. Discussions about pacing were seen as helpful. Discussing their experiences provided a sense of validation.
3. *Did REGAIN make a difference?* Control participants felt they were provided with guidance, they were glad to help others, and appreciated talking to someone who listened. One person felt they were already doing everything suggested so for them it did not make a difference.
4. *Your COVID recovery* (YCR): all control participants were given this resource, freely available online as an NHS resource. Five did not recall getting this resource. One called it ‘a secret resource’ rather than something being freely given out. Six found it useful (two for support and guidance, two were reassured symptoms normal and recovery would be slow and two felt the message they would get better was helpful). Three did not find it useful, two did not take the information in and two found it daunting. Only two people remembered accessing the listed resources at the end of the YCR leaflet.

### Contextual issues

The main contextual issues throughout the data from the intervention group were due to the multiple Information Technology (IT) issues encountered, the changing phases of the pandemic in the UK and its effect on participant experiences and societal attitudes towards people’s recovery from COVID-19.

#### Multiple Information Technology (IT) issues

Delivering exercise sessions remotely was not the norm for practitioners (only one had experienced it before REGAIN) or patients but was necessary due to the pandemic. The training for using the Beam platform, which used Zoom, went well in training. However, the reality in the field during the trial showed many obstacles which hindered the timely delivery of the intervention. Some participants’ understanding and ability to use the IT were limited. These needed extra time and assistance to link up, and some forgot how to log in due to memory problems associated with post-COVID-19 condition. Connection issues included videos not working and ‘drops’ in connectivity. Different devices threw up different problems including not being able to see people exercising, ‘propped up’ smart phones and compatibility problems with differing devices and emails providers. These were stressful and time consuming.

…*And occasionally you’d get somebody who was not so…Au fait with…using the Internet and using zoom…I know some of my colleagues like took 1/2 an hour on the phone before they could get somebody on to the video call…some of the times the questions that were coming back, you know if somebody was trying to access it from their phone, for example, the functionality is quite different. If it’s an iPhone or a Samsung, then it would respond differently. There were quite a lot of connotations between Apple and Windows laptop tablet… we sort of learned as we went, but…And also people with certain e-mail addresses would not get our emails…they wouldn’t even get it in the junk mail*. P2

A second practitioner had been put in place on each group session for safety reasons. If anyone disappeared from a call or became distressed in any way, physically or emotionally, the role of the second person was to take them into a breakout room or talk to them over the phone allowing the sessions to be continued uninterrupted. In reality, this happened on relatively few occasions (although still needed), and the second person was most often needed to troubleshoot IT problems.

#### Participants’ experiences within a pandemic

The course of the pandemic had a changing backdrop of waves with societal impacts (lock downs, social restrictions and rules) but also evolving medical knowledge and treatments. The first wave of those hospitalised had fewer options for treatment, added to unprecedented pressure on clinicians and health services and high hospital mortality rates, with no vaccines on the horizon. Stories of close family deaths and seeing deaths in hospital were common; some reported they had PTSD from their experiences in hospital. Later waves had more treatment choices (eg, dexamethasone), better medical understanding and less invasive treatment options (eg, continuous positive airway pressure) which improved the mortality rate. Most participants described fast discharges with little or no support when they got home. Although they were happy to have survived, most did not receive any guidance in how much or how little to do. This isolation was magnified by lockdown with restricted access to healthcare or support. Many of our participants did not know of others who had been affected similarly to them. Throughout, there was the recurrent fear of catching COVID-19 again even when vaccinated.

*I just thought, ‘Wow, they have been through a hell of a lot.’ But then it was nice as it went on as well to see that as I was doing more and more groups and you were dealing with people who were in the second wave that how their experience was completely different, because new treatments had been found and it meant that there wasn’t the sheer amount of isolation that there was before for people, and they had a completely different experience of their journey throughCOVID.* P3

#### Stigma of societal attitudes towards people’s recovery

Society’s views on COVID-19 were seen through a lens of what they themselves were experiencing. In the initial stages this included fear of the unknown with sudden lockdown, an overwhelmed NHS and a daily death tally. Public opinion often centred around ‘Died or Survived’ with no understanding of any long-term effects even when the term long COVID was used. As time went by the fear of exposure, the relief of vaccination, learning to live alongside COVID-19 and starting to return to normal life led some people to dismiss COVID-19 as ‘just like influenza’. This had the effect of devaluing or negating our participants past COVID-19 experiences, residual symptoms and fears. Some participants reported that they were made to feel they were faking it or at least making more of their symptoms than was the case.

…*I didn’t know whether the kind of general public were aware just to the extent of some people’s suffering… … and I don’t know if this is known more widely*… P2

…*COVID itself is horrendous on top of that and then of course, we have to deal with the world saying, you know, half the world saying it doesn’t exist, the other half saying it’s just flu. You know, some of the people saying, ‘Well, it’s only people, old people who’ve got underlying conditions who…’ …just very coolly and calmly and coldly say that people think that dying is actually the worst thing. But for me, being half alive is worse…it isn’t two weeks of flu and then you’re back at work or you’re dead. There’s a whole range of awfulness in between and I’m in the middle*… Int13

### Practitioner training

All practitioners felt that the training was good, fit for purpose and standardised. They commented on three specific areas.

#### Preparation

They felt prepared and supported especially with the exercise component. The support sessions were an extension of their previous experience, but they felt the training, patient manuals, the practitioner guidance sheets and videos helped them to run and cover effective sessions.

…*I think the training was really detailed and it was really useful and I know we had new people come on further down the line to help us with the delivery and, you know, as far as I’m aware the training didn’t change. We still had the same manual that we all used. All the information was in there*. P3

…*The manual was good. Extremely comprehensive, which was, which is particularly good at the start…those videos, but yeah, they really helped kick off the discussions and you know that made the practitioner job really easy…It helped to have us to sort of guide and stop it from disappearing off too many tangents…* P2

#### Group facilitation

Advice on group facilitation from health psychologists was seen as especially helpful.

*I think all of us on the kind of practitioner team felt like we had some experience of, I suppose the softer side of kind of, I suppose physical activity, counselling and even sort of health education at a sort of fairly low level, but this this was a…a big step up and beyond anything that I’ve done before…So the training that we had from [names of health psychologists] was absolutely vital, I think.* P2

#### Ongoing support

Immediate feedback and access to the whole team were appreciated.

Being in the same central venue allowed more experienced members of the team to support newer members.

*The great thing was I had certainly, because it was the first groups I had some very instant feedback from the rest of the team so, you know, they were online as well as a group watching. There was some really detailed feedback straight away which on the whole was really good*. P3

## Discussion

What have we learned from the REGAIN intervention?

Our key findings were that the support group sessions were valued and enabled participants to feel they were not alone and could share experiences and address worries. The exercise sessions catered for different abilities, and participants reported feeling safe and confident to exercise at a level at which they felt comfortable. The 1:1 consultation sessions revealed patients needed to tell their stories.

Participants and practitioners alike described the REGAIN intervention as a learning experience. Few changes are needed to the original intervention format and content. These changes evolved from working with participants experiencing the effects of an unknown disease presentation, and the findings revealed that the intervention had enough flexibility to incorporate them. Circumstances within which this study was conducted were in some ways unique given the unknown pathology and uncertain prognosis of a new condition of which participants and professionals alike had no experience and for which there was limited empirical data. The prevalent post-hospitalisation post-COVID-19 condition population are likely to face a somewhat different set of circumstances which may affect the benefit derived from the REGAIN programme described in this qualitative study.

The support sessions were a valued aspect of the intervention allowing participants to share their experiences with people who understood and explore their concerns which helped to lessen feelings of fear of activity and isolation. We found that remote exercise was acceptable to patients and practitioners if there were safety measures in place as previously described. Patients and practitioners felt reassured by these measures and gained confidence to explore their exercise capabilities.

It was clear that participants and practitioners were aware that ‘one set programme doesn’t fit all’ due to differing physical abilities, ages, symptoms and presentations. Concurrent multilevel exercise programmes with flexibility to move between levels for each exercise proved feasible and were appreciated by participants. Participants were often apprehensive or fear avoidant about exercising, especially not wanting to overdo it and worsen their symptoms, for example, fatigue and breathlessness. All programmes started at a low level before progressing as the individual was able, ensuring the onus was on them to decide the level they were exercising at, with guidance, and to increase the effort as able/needed.

This qualitative evaluation revealed that online delivery of support and exercise sessions was acceptable. This provides an increased confidence for clinicians and policymakers for future implementation and replicability. There were multiple IT issues which were important to resolve before people attended group sessions, and the 1:1 consultation was important to allow this to happen. We are not aware of any other qualitative evaluations that have been undertaken to increase our understanding of an intervention for post-COVID-19 condition.

It may be that this intervention could improve services for people after, for example, significant trauma, or those undergoing rehabilitation, with the need to tell their story and feel they are not alone as important aspects of care.

### Strengths

This qualitative evaluation gave voice to people taking part in the study, which helped us understand how participants and practitioners experienced support and exercise sessions. The study showed that people with post-COVID-19 condition often needed to tell their story, which could be traumatic, and this also affected the practitioners who heard these stories, and who valued being supported by colleagues. Keeping data collection and analysis completely separate from the main RCT meant the qualitative evaluation findings were made available to the chief investigator before the results of the main RCT were known. This helped protect against possible bias in the qualitative evaluation which could have been introduced if the RCT findings were known.

### Limitations

The timing of this study early in the pandemic meant that it addressed the needs of hospitalised patients from the early days of COVID-19 and post-COVID-19 condition, which may represent a small proportion of the overall current population with post-COVID-19 condition, thus limiting the generalisability of the findings. The design of this qualitative evaluation did not allow for detailed collection of data (eg, undertaking interviews) from non-attendees or those who dropped out, so we do not understand in detail why people did not attend or dropped out of the study. Although the intention was to include participants with a range of interviewee characteristics, 75% of participants reported white ethnicity, thus limiting the reach of the study. In the main RCT, 88% reported white ethnicity. Future research should attempt to ensure a more diverse ethnic mix, involving diverse communities in patient and public involvement and engagement.

## Conclusion

Prior to this qualitative evaluation for the REGAIN RCT, it was not known whether remotely supervised online exercise sessions were feasible, acceptable or well tolerated for participants and practitioners. Some practitioners had initial doubts about this mode of delivery. We now know that offering a programme of online exercise and psychological support run by experienced exercise specialists can be carried out in a way which is acceptable and useful for adults with post-COVID-19 condition, at least 3 months after hospital discharge for COVID-19. Sharing and bonding were in evidence in the groups, where people often compared themselves to others and found discussions with other people ‘in the same boat’ extremely helpful.

## supplementary material

10.1136/bmjopen-2024-085950 online supplemental table 1

10.1136/bmjopen-2024-085950 online supplemental appendix 1

## Data Availability

Data are available upon reasonable request.
