# Supplementary material for: Qualitative evaluation of the Rehabilitation Exercise and psycholoGical support After COVID-19 InfectioN (REGAIN) randomised controlled trial (RCT): ‘you are not alone’
Source: BMJ Open. 2025 Jan 29;15(1):e085950. doi: 10.1136/bmjopen-2024-085950 (PMC11784388; doi:10.1136/bmjopen-2024-085950)
Supplement: online supplemental table 1 [file bmjopen-15-1-s001.pdf]

Supplementary Material

Table S1. Themes presented and source of those themes

Support Sessions themes	Data Source
• You're Not Alone<br>• Sharing Experiences and Addressing Worries<br>• Gaining New Perspectives<br>• Hope for Progression<br>• Peer Support and Bonding	**Participant and practitioner interviews**
• Integration of facilitation skills<br>• Modified activity pacing and goal setting<br>• Giving participant's structure	**Practitioner interviews**
**Exercise Sessions themes**	
• Monitoring and Modification of Online exercise<br>• Catering for differing abilities<br>• Feeling safe and confident to exercise<br>• Progression of Fitness<br>• Optimal timing in the recovery trajectory<br>• Group Effect	**Participant and practitioner interviews**
• Initial apprehension about exercise group<br>• Gauging Exercise Capabilities<br>• Translating Exercises into life<br>• On Demand supplementary videos	**Participant interviews**
**1: 1s Intervention and Control**	
• Traumatic stories needing to be told: General and Control<br>• Practitioner Support<br>• Recovery Trajectory<br>• Using the Your Covid Recovery resource	**Practitioner interviews**
• Trial processes and preference of allocation	**Participant interviews**

• Experiences<br>• Did Regain make a difference?<br>• Your Covid Recovery Resource	
**Contextual Issues**	
• Multiple IT issues<br>• Participants' experiences within a pandemic<br>• Stigma of societal attitudes towards people's recovery	**Participant and practitioner interviews**
**Practitioner Training**	
• Preparation<br>• Group Facilitation<br>• Ongoing Support	**Practitioner interviews**
