# Supplementary material for: Qualitative evaluation of the Rehabilitation Exercise and psycholoGical support After COVID-19 InfectioN (REGAIN) randomised controlled trial (RCT): ‘you are not alone’
Source: BMJ Open. 2025 Jan 29;15(1):e085950. doi: 10.1136/bmjopen-2024-085950 (PMC11784388; doi:10.1136/bmjopen-2024-085950)
Supplement: online supplemental appendix 1 [file bmjopen-15-1-s002.pdf]

Appendix 1a – Indicative Interview Prompts for Patient Participants

Introduce self. *You have been helping with the REGAIN study and we are interviewing some participants to see what they thought about participating. Did you have any questions about the study or this interview?*
You have already helped a great deal by being in the study, these interviews will help us to understand more about peoples' experiences of being on the study. Can I confirm that you are happy for this interview to be recorded? (Go through consent form and record separately) Interviews usually take under an hour and you can halt it at any time.

<u>Introduction (All):</u>

How did you hear about the REGAIN study?
How long ago were you admitted into hospital with COVID-19?
How long were you in hospital?

1) ***Please can you tell us about your experiences of taking part in this study?***
   General prompts: Please can you say a bit more about that; how did that make you feel; what did you do next? Was there anything that helped? Was there anything that was more challenging?

<u>For patients in intervention arm</u>

2) ***How was the 1:1 session with the practitioner?***
   Prompt: Was anything helpful, was anything more challenging?
3) ***How were the exercise sessions?***
   Prompt: How did you feel about doing this at home? (Challenges, enablers)
4) ***How were the demand sessions?***
   Prompt: Exs Br Exs Pilates Yoga
5) ***How were the group support sessions?***
   Prompt: 1. Motivation goal setting 2. Activity avoidance 3. Pacing activity and tackling fatigue 4. Sleep behaviour 4. Managing emotions, mood and thoughts, 6. Managing setbacks and future goals.
   Did anything help? Was anything more challenging?
6) ***What did you think about the workbook?***
   Prompt: Anything useful / not useful

<u>For patients in control arm</u>

2) ***How was the 1:1 advice session?***
   Prompt: Was anything helpful, was anything more challenging?
3) ***How did you get on with what was suggested?***
4) ***Were you able to access the resources suggested?***
   Prompt: Your COVID recovery Book/Link to, what was useful/ not useful
5) ***How did you find the resources suggested?***

<u>All</u>

6) ***How were the online trial processes?***
   Online enrolment /Questionnaires/Randomisation/Preference
7) ***What advice would give to someone thinking of starting the programme you had?***
   General prompts: Please can you say a bit more about that; how did that make you feel; what did you do next?
8) ***What's the plan from now?***

9) ***Is there anything else you think I need to know?***
10) ***Is there anything else you'd like to say that I haven't asked you about?***

Thank participant for helping with the REGAIN study and the interviews.

Appendix 1b. ***REGAIN practitioner (intervention delivery staff) interview: Indicative interview prompts***

Introduce self. *This interview is to see what you thought about the training and delivery of the REGAIN study interventions. Do you have any questions from the information sheet we sent or the consent form you signed?*
Are you happy for this interview to be recorded? (Go through consent form if necessary and how the audio recordings are transcribed and anonymised) Interviews usually take under an hour and you can halt it at any time. These interviews are confidential and individuals will not be named.

1) **Could you begin by giving me a few details about yourself? Your job title, years qualified. How did you come to work on the REGAIN study?**

2) **Tell me about your experience of being a REGAIN practitioner in this study.**

3) **How did you find the training?**
    a) **Positive things? Things to improve?**
    b) **How was the manual?**
    c) **How were the video clips?**

4) **Did you do any of the initial 121's? Tell me about those.**

5) **How did you find supervising exercise practice (recorded and live)?**
    a) Anything that helped; anything that hindered? Any concerns?
    b) How did you feel about doing this before you started?
    c) How did you feel about doing this once you got started?

6) **What was your experience of delivering the psychosocial/motivational aspects of the intervention?**
    a) **Anything that helped; anything that hindered? Any concerns?**
    b) **How did you feel about doing this before you started?**
    c) **How did you feel about doing this once you got started?**

7) **What advice would you give to a practitioner thinking about delivering this intervention?**

8) **Is there anything else you'd like to say that I haven't already asked you?**

Thank you so much for taking part in this interview study.
