# Supplementary material for: Qualitative evaluation of the Rehabilitation Exercise and psycholoGical support After COVID-19 InfectioN (REGAIN) randomised controlled trial (RCT): ‘you are not alone’
Source: BMJ Open. 2025 Jan 29;15(1):e085950. doi: 10.1136/bmjopen-2024-085950 (PMC11784388; doi:10.1136/bmjopen-2024-085950)
Supplement: Supplementary file 3 [file bmjopen-2024-085950.reviewer_comments.pdf]

PEER REVIEW HISTORY

BMJ Open publishes all reviews undertaken for accepted manuscripts. Reviewers are asked to complete a checklist review form and are provided with free text boxes to elaborate on their assessment. These free text comments are reproduced below.

ARTICLE DETAILS

Title (Provisional)

A qualitative evaluation of the Rehabilitation Exercise and psycholoGical support After covid-19 InfectioN (REGAIN) RCT: “You’re not alone”.

Authors

Seers, Kate; Nichols, Vivien P; Bruce, Julie; Ennis, Stuart; Heine, Peter; Patel, Shilpa; Sandhu, Harbinder Kaur; Underwood, Martin; McGregor, Gordon

---

VERSION 1 - REVIEW

**Reviewer**	**1**
**Name**	**Wade, Derick**
**Affiliation**	**Oxford Centre for Enablement**
**Date**	**20-Mar-2024**
**COI**	**None**

Conclusions of this review.
The paper needs to be completely rewritten with a proper introduction, sufficient description of the methods for the study to be replicated, full explanation of patient flow through the main study and the qualitative study, explanation of the analysis, and presentation of themes much more succinctly with much less production of quotations. In the results section, around 80% should be plain text, with 20% being supporting quotations. Existing guidelines should be followed. The discussion should not add new information.

If this qualitative study is based on data from less than half of the 40 people approached, then this needs to be made evident in the abstract.

REVIEW

The title is clear.

The abstract is informative and precise. It would help to know how many people were in the complete study and how these 40 patients were selected from the larger group to be certain it is a reasonably representative sample. The conclusion could be shortened if the word count is limited.

This was written before reading the paper; in fact the abstract seem quite inaccurate.

Introduction.
I think it is unnecessary to outline, albeit briefly, the history of the pandemic. The introduction could start with "the term long Covid".

The second paragraph should, in my view, be part of the methods section. The introduction needs to set this in the context of any other studies in this condition and, potentially, studies of other similar conditions such as fibromyalgia. This is an editorial decision.

Methods.
The methods should set this in the context of the particular trial. There should be information on its registration number, ethics committee permission reference number, and organisation responsible for its integrity and conduct. The start date and end date of the whole study and of this study should be given. In this part, the reference to previous publication should be given rather than in the introduction.

What is "Atrium Health"? Although details are published elsewhere, sufficient details need to be given here for the reader to know all the important facts. Was it a multicentre trial? I assume that the Impact of Event Scale was collected as part of the main trial. This should be explained.

There are insufficient details about the thematic analysis to allow evaluation. How many people were involved? What cross-check was there? Were emerging themes discussed with the participants?

I am not an expert in qualitative research, but I think that the authors should adhere to the standards for reporting qualitative research and the recommendations or the consolidated criteria for reporting qualitative research. (COREQ)

The method section needs huge improvement.

Results.
I think the data should also include data from the total sample to show how these 40 people relate to them. It is unclear what the hospital care setting means. They were interviewed at home. Was this the highest level of dependency achieved while in the hospital? Although the numbers are fairly small, I think it would be reasonable to provide the mean and standard deviations for age and scores on the two measures used.

It is not clear how many people were interviewed. Halfway through the results, we are informed that 18 participants did not proceed with interviews, 15 of which were

control participants. If so, this implies that only five control patients were interviewed.

If I have misunderstood this, then the authors need to reconsider their whole results section to clarify matters.

In contrast, the results are presented in too much detail. It is difficult to see what the themes are, as they are overwhelmed with quite long quotations. Most qualitative studies I have read, and that is a large number, have presented data much more succinctly. The authors need to do more work on presenting the data in text, with smaller quotes to illustrate the point. If they wish to give long, extensive quotations, they can be supplementary data.

The table on fidelity is almost impossible to interpret because we have been given no information on how fidelity is recorded. Percentages are often misleading, and the numbers generating the percentage need to be presented.

Discussion.
This adds new data. I do not understand the relevance of 40 not attending the intervention group and 28 not attending the control group. Is it related to the whole study?

Conclusions of this review.
The paper needs to be completely rewritten with a proper introduction, sufficient description of the methods for the study to be replicated, full explanation of patient flow through the main study and the qualitative study, explanation of the analysis, and presentation of themes much more succinctly with much less production of quotations. In the results section, around 80% should be plain text, with 20% being supporting quotations. Existing guidelines should be followed. The discussion should not add new information.

If this qualitative study is based on data from less than half of the 40 people approached, then this needs to be made evident in the abstract.

**Reviewer**	**2**
**Name**	**Décary, Simon**
**Affiliation**	**University of Sherbrooke**
**Date**	**22-Apr-2024**
**COI**	**N/A.**

This paper presents a process evaluation of the REGAIN study, a randomized trial focused on exercise and psychological support for individuals with Long COVID. As someone who has been involved in Long COVID research since the start of the COVID-19 pandemic, I was

already familiar with this trial, including the lukewarm reception it received from some patient organizations on social media. This negative response likely contributed to recruitment challenges (this study is from 2021). I was excited to finally read the full publication of the main results in BMJ a few weeks ago, although these results also received pushback from advocacy groups. Given this history, I believe that this process evaluation is critical to providing evidence-based responses to criticisms. Notably, this appears to be the first and only paper on exercise in post-covid-19 condition (PCC) (out of hundreds) to have conducted a process evaluation to explain the findings of a randomized controlled trial. The paper serves as a valuable reference for future RCTs in the field of post-infectious conditions, illustrating the best methodological approaches for studies incorporating process evaluations. However, the paper has several shortcomings.

The main criticism and limitation is that this paper addresses the needs of post-hospitalized patients from the early waves of the pandemic. This subgroup represents less than 10% of the overall population with PCC, and evidence suggests that their condition may resemble post-intensive care syndrome more than typical Long COVID such as the lower prevalence of PEM. I suggest that the authors frame the paper in terms of "post-discharge" rather than Long COVID, as has been done in previous publications in the British Journal of Sports Medicine. The authors should also have better contextualized their intervention for this population, as recent systematic reviews differentiate between hospitalized and non-hospitalized groups. Providing more detailed information on existing rehabilitation programs and psychological support approaches would help situate the REGAIN study within the broader research landscape. This reframing could address many of the criticisms regarding the representativeness of the population and the relevance of the results to PCC patients. Additionally, the characteristics table should include the number of weeks of symptoms when receiving the intervention and which wave of the pandemic they were a part of. It is crucial not to overstate the scope of the REGAIN study, but to frame it accurately for what it is.

While the above points are relatively straightforward to address, I am concerned that the paper might not meet the qualitative research standards for BMJ Open. Since most results are based on interviews, I expected the manuscript to follow appropriate reporting guidelines. The methods section is lacking in detail. There is no information on the development of the interview guide, and it is not available as supplementary material. This omission raises questions about the interview content and process, which are critical for understanding the results. The analytical framework for the qualitative data is also unclear, as inductive thematic analysis should be described in more detail in BMJ Open. The sample size determination and recruitment process could be explained in more detail to ensure replicability. Additionally, the reporting of the qualitative results is confusing, with an overreliance on multiple quotations. This approach makes the manuscript challenging to read, and a more structured presentation would be beneficial. I would prefer to see this

section framed in the context of current PCC rehabilitation research and how the data answer questions about rehabilitation interventions (e.g., focus on safety issues).

Overall, while the paper has potential, it may not meet the required standards for BMJ Open without significant revisions.

VERSION 1 - AUTHOR RESPONSE

**Reviewer 1**		
1.1)	The paper needs to be completely rewritten with a proper introduction, sufficient description of the methods for the study to be replicated, full explanation of patient flow through the main study and the qualitative study, explanation of the analysis, and presentation of themes much more succinctly with much less production of quotations. In the results section, around 80% should be plain text, with 20% being supporting quotations. Existing guidelines should be followed. The discussion should not add new information.	This paper has been rewritten in line with the helpful comments from both reviewers. We have reduced the findings section considerably and removed all the tables which contained multiple quotations so it is much more succinct. Although this is a mixed methods study, we have adhered to the COREQ guidance for qualitative studies amd include a completed COREQ template with this submission.
1.2)	If this qualitative study is based on data from less than half of the 40 people approached,<br>then this needs to be made evident in the abstract…. It would help to know how many people were in<br>the complete study and how these 40 patients were selected from the larger group to be certain it is a reasonably representative sample.	We have made it clearer that the study is based on data from 20 people in the intervention group and 20 in the control group. The way in which these 40 were selected from the larger group is detailed in the methods (p4 and p7), our aim being to have a diverse sample.
1.3)	**Introduction**.<br>I think it is unnecessary to outline, albeit briefly, the history of the pandemic. The introduction could start with "the term long Covid"… The introduction needs to set this in the context of any other studies in this condition and, potentially, studies of other	We have removed the sentence that outlines the history of the pandemic, but left the sentence that identified the size of the problem of post covid condition. (p3). We have not included fibromyalgia, as this is not the focus of this study.

	similar conditions such as fibromyalgia. This is an editorial decision.	
1.4)	The second paragraph should, in my view, be part of the methods section	We have discussed this and argue it is more coherent to include this second paragraph in the introduction as it sets the scene for the process evaluation, rather than detailing the methods of the wider RCT.
1.5)	**Methods**<br>There should be<br>information on its registration number, ethics committee permission reference number,<br>and organisation responsible for its integrity and conduct. The start date and end date of the whole study and of this study should be given. In this part, the reference to previous publication should be given rather than in the introduction.	Information on the ethical approval number and registration reference and study research sponsor is supplied. (p24).<br><br>Start and end date of overall study and of the process evaluation have been added (p6- beginning of results section).
1.6)	What is "Atrium Health"?	This has been added (p4)
1.7)	Although details are published elsewhere, sufficient details need to be given here for the reader to know all the important facts. Was it a multicentre trial? I assume that the Impact of Event Scale was collected as part of the main trial. This should be explained.	Thank-you for this comment, we have revised the text to make this clearer. P4.
1.8)	There are insufficient details about the thematic analysis to allow evaluation. How many people were involved? What cross-check was there? Were emerging themes discussed with the participants?	The thematic analysis section has been revised to make this clearer. P5-6
1.9)	the authors should adhere to the standards for reporting qualitative research and the recommendations or the consolidated criteria for reporting qualitative research. (COREQ)	This is a mixed methods study, but we have also included a completed COREQ with this submission.
1.10)	**Results**.<br>I think the data should also include data from the total sample to show how these 40 people relate to them. It is unclear what the hospital care setting means. They were interviewed at home. Was this the highest level of dependency achieved while in the hospital? Although the numbers are fairly small, I think it would be reasonable to provide the mean and standard deviations	Thank-you for this comment which we have discussed carefully. We argue that as we are trying to be concise, this is not needed in this process evaluation where we have purposively selected participants in order to have a diverse sample, as explained in the methods section, and have clearly

	for age and scores on the two measures used.	presented this in Table 1 of the findings. (p7)
1.11)	It is not clear how many people were interviewed. Halfway through the results, we are informed that 18 participants did not proceed with interviews, 15 of which were control participants. If so, this implies that only five control patients were interviewed. If I have misunderstood this, then the authors need to reconsider their whole results	Thank-you for identifying that this was unclear – we have made this clearer in the findings. We did interview 20 participants in the intervention and 20 in the control group. The 18 referred to are participants who said during the main trial that they would be happy to be approached, but when approached declined to take part in the process evaluation interviews. P7
1.12)	The results are presented in too much detail. It is difficult to see what the themes are, as they are overwhelmed with quite long quotations. Most qualitative studies I have read, and that is a large number, have presented data much more succinctly. The authors need to do more work on presenting the data in text, with smaller quotes to illustrate the point. If they wish to give long, extensive quotations, they can be supplementary data.	We have taken this helpful comment on board and completely revised the findings section, making it much more succinct and we think it is now much more accessible. P8-20
1.13)	The table on fidelity is almost impossible to interpret because we have been given no information on how fidelity is recorded. Percentages are often misleading, and the numbers generating the percentage need to be presented.	The fidelity section has been revised. It needs to be viewed alongside the supplementary material 1 where the templates for fidelity (adherence and competence) are available. They are not included in the main text because they are so detailed.
1.14)	Discussion.<br>This adds new data. I do not understand the relevance of 40 not attending the intervention group and 28 not attending the control group. Is it related to the whole study?	We agree this was unclear and have rewritten this section p21-24
**Reviewer 2**		
2.1)	I believe that this process evaluation is critical to providing evidence-based responses to	Thank-you for your positive comments.

	criticisms. Notably, this appears to be the first and only paper on exercise in post-covid-19 condition (PCC) (out of hundreds) to have conducted a process evaluation to explain the findings of a randomized controlled trial. The paper serves as a valuable reference for future RCTs in the field of post-infectious conditions, illustrating the best methodological approaches for studies incorporating process evaluations.	
2.2)	The main criticism and limitation is that this paper addresses the needs of post-hospitalized patients from the early waves of the pandemic. This subgroup represents less than 10% of the overall population with PCC	We agree that this paper addresses the needs of post hospitalised, some of whom are from the early waves of the pandemic. This has been added to the discussion section, p22.
2.3)	I suggest that the authors frame the paper in terms of "post-discharge" rather than Long COVID, as has been done in previous publications in the British Journal of Sports Medicine. The authors should also have better contextualized their intervention for this population, as recent systematic reviews differentiate between hospitalized and non-hospitalized groups. Providing more detailed information on existing rehabilitation programs and psychological support approaches would help situate the REGAIN study within the broader research landscape. This reframing could address many of the criticisms regarding the representativeness of the population and the relevance of the results to PCC patients.	Having discussed this we agree and have used the term “post covid condition”. There is some use of the term “long covid” by patients and this has been retained.<br>The rest of this comment refers to the main RCT study rather than the process evaluation.
2.4)	The characteristics table should include the number of weeks of symptoms when receiving the intervention and which wave of the pandemic they were a part of	We have included extra data about meantime from hospital discharge. P7.
2.5)	It is crucial not to overstate the scope of the REGAIN study, but to frame it accurately for what it is.	We have given more details about the context so the scope is clearer and we have been careful not to overstate the scope of REGAIN. See also 2.3
2.6)	I am concerned that the paper might not meet the qualitative research standards for BMJ Open. Since most results are based on interviews, I expected the manuscript to follow appropriate reporting guidelines.	We initially did not include the qualitative reporting guidelines, as this is a mixed methods study. However, given the reviewer’s concerns about details of the methods, we have addressed this

		concern by including qualitative reporting guidelines COREQ to reassure you we have followed the appropriate guidelines and this meets qualitative research standards.
2.7)	The methods section is lacking in detail. There is no information on the development of the interview guide, and it is not available as supplementary material	We have extended the section on development of the interview guide, and have included the questions in appendix 1. P28-29
2.8)	The analytical framework for the qualitative data is also unclear, as inductive thematic analysis should be described in more detail	The thematic analysis is now described in more detail (p5)
2.9)	The sample size determination and recruitment process could be explained in more detail to ensure replicability	The recruitment process is now explained in detail in the methods section. (p4-6). The sample size determination for the process evaluation will depend on when the data becomes saturated for an understanding of the theme. This will vary between studies.
2.10)	The reporting of the qualitative results is confusing, with an overreliance on multiple quotations. This approach makes the manuscript challenging to read, and a more structured presentation would be beneficial	We agree and have made the findings much more succinct, see also 1.12
2.11)	I would prefer to see this section framed in the context of current PCC rehabilitation research and how the data answer questions about rehabilitation interventions (e.g., focus on safety issues).	This is now framed in the discussion (p21-24).

VERSION 2 - REVIEW

Reviewer 1

Name Wade, Derick

Affiliation Oxford Centre for Enablement

Date 15-Sep-2024

COI

I have answered 'no' to most of the questions because the part of the paper concerned with fidelity is weak, with minimal description or data, and should be removed. I would have said yes to the qualitative part of the paper.

The paper's description of the qualitative study of the experiences of patients and professionals is much improved. Minimal information is given about the process evaluation (i.e. the quantitative measures of adherence to the specified intervention), and this should be removed and covered in a separate paper.

This is a much-improved version of the paper. Nonetheless, I have some comments that the authors should consider. I also note that much data is in the supplementary data file, but this was not provided, so I cannot comment on it.

The term process evaluation may not have an absolute definition, but it is associated with establishing fidelity, giving data on how closely the intervention adhered to the expected protocol. It is largely a quantitative matter. There may be some data on fidelity, but it is not available in the paper submitted for review, nor is the associated method description.

This paper is qualitative focused primarily on the experiences of patients and professionals.

Consequently, the title and the abstract must emphasise that this is a qualitative study of patients and professionals. This may require some adjustment to the background and objective in the abstract, as well as a significant change in the title. At present, anyone searching for a qualitative study on the experiences of patients and professionals delivering rehabilitation would not find this paper.

Next, the paper alluded to but did not draw attention to the unique circumstances of this study, which affected the experiences of both patients and professionals. They were faced with a radically new condition about which little was known and with an uncertain prognosis. In almost all other health conditions, much is known about the condition, at least by the professional. Some of the comments allude to this directly.

This should at least be mentioned when interpreting and discussing the data. This fact also potentially limits the interpretation or use of the data. Much is now known about the condition, and there is little in the way of shared experience between patients and professionals. The authors might set this in the context of the response to the influenza epidemic after World War I.

Third, the studied patient population is selected by virtue of being admitted to the hospital. This again limits generalisation. Patients were inevitably selected because they were part of the REGAIN study. This does need to be mentioned as a limitation.

The fidelity aspect of this study should not be included in this paper. The paper contains minimal information about the methodology. Furthermore, the data were not provided for me to review, nor could I review the methodology.

In keeping with my comments on process evaluation, the authors should separate this paper into two components. This paper is a qualitative study of the experiences of patients and professionals delivering and receiving the intervention. A second paper should be concerned with the fidelity of the intervention's delivery, which is a different topic.

VERSION 2 - AUTHOR RESPONSE

The paper's description of the qualitative study of the experiences of patients and professionals is much improved. Minimal information is given about the process evaluation (i.e. the quantitative measures of adherence to the specified intervention), and this should be removed and covered in a separate paper.

Thank you for the suggestion – we have now removed all assessment of fidelity from the paper.

This is a much-improved version of the paper. Nonetheless, I have some comments that the authors should consider. I also note that much data is in the supplementary data file, but this was not provided, so I cannot comment on it.

We are not quite sure why you couldn't access the supplementary materials. They are included on the system and appeared in the system generated PDF. Irrespective, the file has been amended now to remove all the process evaluation data. An amended supplementary material has been added including only the themes generated form the interviews.

The term process evaluation may not have an absolute definition, but it is associated with establishing fidelity, giving data on how closely the intervention adhered to the expected protocol. It is largely a quantitative matter. There may be some data on fidelity, but it is not available in the paper submitted for review, nor is the associated method description.

All reference to the assessment of fidelity has been removed from the paper.

This paper is qualitative focused primarily on the experiences of patients and professionals. Consequently, the title and the abstract must emphasise that this is a qualitative study of patients and professionals. This may require some adjustment to the background and objective in the abstract, as well as a significant change in the title. At present, anyone searching for a qualitative study on the experiences of patients and professionals delivering rehabilitation would not find this paper.

All sections amended accordingly.

Next, the paper alluded to but did not draw attention to the unique circumstances of this study, which affected the experiences of both patients and professionals. They were faced with a radically new condition about which little was known and with an uncertain prognosis. In almost all other health conditions, much is known about the condition, at least by the professional. Some of the comments allude to this directly.

This should at least be mentioned when interpreting and discussing the data. This fact also potentially limits the interpretation or use of the data. Much is now known about the condition, and there is little in the way of shared experience between patients and professionals. The authors might set this in the context of the response to the influenza epidemic after World War I.

We have added additional comment with regards to this point. Thank you.

Third, the studied patient population is selected by virtue of being admitted to the hospital. This again limits generalisation. Patients were inevitably selected because they were part of the REGAIN study. This does need to be mentioned as a limitation.

We have been very clear throughout the manuscript (and in related publications) that the REGAIN trial tested an intervention in people hospitalised with covid-19 who continued to experience physical and mental health sequalae beyond 3 months post-discharged. We have described in the limitations sections at the start and end of the manuscript that findings are, therefore, not generalisable to the whole long-covid population.

The fidelity aspect of this study should not be included in this paper. The paper contains minimal information about the methodology. Furthermore, the data were not provided for me to review, nor could I review the methodology.

All mention of fidelity has now been removed.

In keeping with my comments on process evaluation, the authors should separate this paper into two components. This paper is a qualitative study of the experiences of patients and professionals delivering and receiving the intervention. A second paper should be concerned with the fidelity of the intervention's delivery, which is a different topic.

Agreed and actioned.

VERSION 3 - REVIEW

**Reviewer**	**1**
**Name**	**Wade, Derick**
**Affiliation**	**Oxford Centre for Enablement**

Date **12-Oct-2024**

COI

This is well-revised but lacks punch in the discussion. It could be accepted as is, but I fear it will look like "they needed to do it, but it is irrelevant now" whereas much could be taken from this to improve all rehabilitation services.

The paper is much improved. I have no comments or suggestions on the abstract, introduction, methods or results.

The authors have opened up an opportunity to discuss the broader perspectives of their experience. The pandemic was unusual in that a huge number of people fell acutely ill with a life-threatening illness. There are questions the authors may have views on and may have talked about that would be of interest. The discussion is short and gives no sense of excitement or curiosity.

For example, can anything in this be used to improve services for people after a significant trauma such as a motorcar accident occurring to an individual? Are we overlooking something important in people who are acutely ill?

Another area of interest is how we should prepare for the next pandemic. What preparations do the authors think should be undertaken? Given the low likelihood that anyone will prepare, they could develop a list of things a health service should do as soon as the next pandemic starts.

After reading the paper, I feel there is an element of "so what?" The pandemic is over, and the specific circumstances will hopefully not recur for a good few years.

Therefore, it would be interesting to know what general lessons one might draw from this for rehabilitation services. Otherwise, the paper might be dismissed as somewhat interesting but irrelevant.

VERSION 3 - AUTHOR RESPONSE

Thank-you for your comments. We note that reviewer one feels the paper could be accepted as is.

However, they provide additional comments on the discussion section and we have addressed some of these briefly in the discussion.

We have not chosen to develop a list of things the health service should develop as soon as the next pandemic starts, as the study was not designed to answer that question and we do not feel it could provide a robust answer
